# Effect of the act on promotion of personal autonomy and care for dependent persons on their family caregivers

**DOI:** 10.1186/1472-6963-12-462

**Published:** 2012-12-17

**Authors:** Magdalena Cuevas Fernández-Gallego, José Miguel Morales Asencio, Francisco Javier Martín Santos, Rafaela Cruz Arándiga, Eugenio Contreras Fernández, Juan Pedro Batres Sicilia, Francisco Javier Navarro Moya, Isabel Lorenci Abajo, Miriam Celdrán Mañas, Candela Bonill de las Nieves

**Affiliations:** 1 District Health Department of Malaga, Andalusian Department of Health Services, Malaga, Spain; 2 Faculty of Health Sciences, University of Malaga, Malaga, Spain; 3 District Health Department of Northeast Jaen. Andalusian Department of Health Services, Jaen, Spain; 4 District Health Department Costa del Sol. Andalusian Department of Health Services, Malaga, Spain; 5 Andalusian Department of Health Services, Hospital Torrecardenas, Almeria, Spain; 6 Andalusian Department of Health Services, Regional University Hospital Carlos Haya, Malaga, Spain

**Keywords:** Family caregivers, Home care, Gender, Vulnerable populations

## Abstract

**Background:**

The need of home care services is becoming an increasingly common scenario. These cares are mainly provided by the dependents’ relatives specifically, by the women part of the family. This situation might take years, decreasing the physical and psychological health of the caregiver. In Spain, the Act of Promotion of Personal Autonomy and Care for dependent persons, guarantees those dependent persons and their caregivers to have access to social services or to financial grants. The aim of this study is to Know the possible effects of the benefits provided by this Act in regards to the mental health, the quality of life and use of health services by the family caregivers assisting their relatives in situation of dependency.

**Methods and design:**

A longitudinal descriptive study following-up a cohort of patients and caregivers. The study shall be carried out in Andalusia. It shall include the baseline assessment of the variables in those caregivers free from the exposure factor (reception of assistance pursuant to the Act). Following, once the benefits have been received, this cohort shall be followed-up.

The study shall take three years, and the starting date for its development as well as its funding is January 2011.

**Discussion:**

The longitudinal assessment of the rate of change of the variables studied shall allow us to know the implications which might be potentially generated as well as the natural evolution of those.

## Backgrounds

Our society is nowadays immerse in the adaptation to a new framework derived from social-demographic, epidemiological and cultural determining factors which have led to a progressive inflation of the need of home care services. The composition and operability of the families as well as the gender influence in the family function, are considered as essential elements in regards to the need of home care services. The commitment for providing care is guided by strength cultural standards which generate important driving forces [1], as the family is considered as a matrix cultural value in terms of sustainability. Elderly persons are aware of the difficulties for their descendants to reconcile their socio-labour life with the attention they require, however, despite this fact, is not just a matter about the strong family ties, but also about the bond with the environment in which this relation is developed, thus, considering the strength ties to their home as critical factors in terms of stability within the aging process. Thus, the measures favouring that the elderly persons stay at their home are very appreciated (Day Care Centres, telecare, home care assistance), as they keep an essential pillar in their value system, acting as antidote against loneliness as well as promoting the social participation. In contrast, the institutionalization in elderly nursing homes is negatively considered by society [2].

The scenario shows an inexorable reality: in our country, most of care to dependents persons is provided by non-professionals carers (nearly 90%) [3]. Given the recent data, according to the report issued by the Andalusian Statistics Office (IEA as per its Spanish acronyms) in regards to Dependency and Solidarity in the family net, one third of the Andalusian population has any relative with need of care and assistance and more than half of these people, provides this assistance [4]. This situation contrasts with the situation in those countries with more tradition and development of home care services, as it was highlighted in the study *AdHOC* which carried out a transversal analysis on the different modalities and addressees of home care assistance services in 11 European Countries [5].

Thus, in those countries in the North of Europe, despite the proportion of elderly people living alone is higher, the formal assistance and home care services doubles those services provided in countries in the South of Europe. In regards to the care and assistances assumed by the members of the families at their homes, attending the need of assistance, there are three main type of services: a) Those covering the most basic needs: accommodation, feeding, cleanness, security, etc., b) Those non-waged care directly related to the illness, the disability and the death (early diagnostic, to make company to the patient, transportation, treatment application or follow-up, etc.) and c) the management of the health services use (election, arrangements, payments, information, etc.) [6].

When appointing the roles to provide care among the members of the families, there is a clear gender bias in place, which arises from cultural values ties, being the woman closer to the patient (wife, mother, daughter in law, daughter…) who plays the main role in the care to the patient. Furthermore, this role is usually assigned to those people within the family unit with non-waged activities, and in those cases when the person assigned has indeed a labour activity, they face great difficulties to go ahead with their tasks without suffering a financial negative impact. The recent studies carried out in Andalusia reports about 30% of women devoting more than 20 hours per day to care a loved one [3,7].

According to the report on Living Conditions of Elderly Persons, 57% of the caregivers do not count on anyone else’s support to provide such care and 62% receive a low rate of social support [2]. One review by Morris in regards to 45 studies on home care assistance services and caregivers from a gender point of view, does confirm the aforementioned matter, above all, because men and women do have different social and financial contexts and expectations on their role, and for such reason, women perform situations which requires deeper care assistance. It also identifies a lack of studies comparing the consequences of those choosing the role of family caregiver versus those who are obliged to comply with this role [8].

Durán considers the amount of caregivers in Spain is around 1,500,000 and, according to the author of this study, this amount would correspond to a “working population” rate higher than the total amount including the agricultural, extractive industries, gas and electricity sector [9].

The dependent’s relative becomes his/her caregiver within a progressive process, starting usually by establishing some limits that the caregiver is not willing to exceed (whether due to a lack of capacity or willingness). However, those limits are exceeded insofar the request of care rises. This process, which might take years, decreases the physical health (inappropriate spaces for care, mobilization without help…), and the mental health (mental burden full of uncertainty, anxiety, insecurity, responsibility, etc.…) in those persons performing this role and it often generates social impoverishment (loss of social relationships, difficulties to go ahead or starting labour activities, impact on the relationship with the remaining members of the family) and an irrevocable decrease in the quality of life [10]. The influence of the care on the person while the caregiver performs this function is quite analysed in the respective literature, although not always with solid and conclusive designs. In global terms, the depression appears as the most determining factor of the physical health of the caregivers comparing it with the general population. This fact appears in higher rates in persons caring patients with dementia or behaviour disorders [11,12].

The combination of an extended stress situation, the physical demands of the caring actuations and a higher biological vulnerability in elderly persons who are caregivers themselves, might increase deeply their health problems leading them to death. The standard study of cohorts by Schulz and Beach showed that the mortality risk increased in the elderly spouses who acted as caregivers and experienced tiredness and overburden in regards to this role, thus, being considered as an independent predictor [13]. Later on, some results have been published showing that taking care of a loved one more than 9 hours per week, nearly duplicates the risk to suffer cardiovascular events [14].

Although the emotional disorders and psychiatric morbidity of caregivers have been widely analysed in most of the studies and its remarkable appearance is obvious, there are no suitable studies of cohorts carried out, and, in contrast, there are a huge number of transversal studies.

For instance, in regards to the anxiety, a recent systematic review could not determine which risk factors are considered as the most relevant for the appearance of anxiety in caregivers due to the mentioned lack of methodology [15].

In the depression cases, there are indeed longitudinal studies which have evidenced the long-term impact of the presence of recurrent depression symptoms in caregivers [16]. According to different studies whose results varies significantly, it is recorded an increase of the relative risk of depression ranging from 2.80 – 38.60 [17]. In regards to the quality of life, despite it is obvious that it might be affected by the fact of taking care among those who have lived this experience, and despite there are a number of instruments intended for its assessment with good psychometric properties [18,19], the studies have been mainly focused in the measurement of the overburden which are not considered to be equivalent concepts, although it is related with the quality of life [20].

Given this situation, which are the interventions available? During these last two decades, the number of experimental studies intended to evaluate the different interventions have rose considerably. Many of them are based on cognitive and behavioural interventions, others have an organizational nature, and also multicomponent and pharmacological interventions, etc. In the last 6 years it has been published nearly a tens of meta-analysis and systematics reviews which have assessed many of these aspects [21,22]. In general terms, some behavioural interventions whether in group or individually have effects on the satisfaction, the subjective welfare, the depression or the knowledge level. However, the heterogeneity of studies and designs encourage to study these interventions in-depth in prospective research of better quality, including a higher number of patients as well as with a better definition of the interventions. In summary, the multicomponent intervention and specifically those involving the patient are usually the most effective interventions. It is worthy to mention that the respite care, despite it improves the satisfaction, does not affect the overburden and they neither reach to postpone the institutionalization [23,24].

Besides, the results of qualitative research are quite numerous in the specialized literature and have described the experience of the informal care in different contexts and aspects, however, for the time being, there are no qualitative meta-synthesis which might allow to achieve evidences [25,26]. The fact that the Health Services address their attention to this these caregivers increases the opportunities of encounter as well as it improves the sensitivity of the health professional sector about the specific situation they live. In the research study by Morales, which was carried out in Andalusia, it has been evidenced how the intervention under a model of cases management, substantially improved in 12 months the overburden level in the experimental group. It likewise improved the accessibility to the social services, improved the multi-professional intervention on the caregivers and their relatives as well as it increased the technical assistance flow, thus, improving their satisfaction while the visits to the Health Centre decreased [7]. Finally, the disposal of measures including benefits and allowances intended for the care of dependent persons have shown an increase in the costs, however, in consideration of these costs, in some studies it has been evidenced an increase of the welfare of both, the dependent person and the caregiver despite there are still some doubts in regards to the assessment methods of these programs in U.S.A. [27]. The German funding model on Dependency has shown positive results as per the increase of community services and the support to the caregivers [28], although it has not reached to solve the permanent distance between the health and social assistance services, among other problems [29]. In other places as it is in Japan, it has been observed how the use of the resources and assistances intended for the dependency are highly influenced by the decisions of the family caregiver [30] and it has been observed some effects of this regulation on the benefits to the dependency on the overburden and depression level of the caregivers [31]; A recent longitudinal study carried out in the mentioned country has shown a lower mortality rate in dependent men who receive informal support, despite this effect was not observed in those cases in which it was the wife who received the benefits for dependency [32].

In Spain, the Act 39/2006 dated December 14^th^, on Promotion of Personal Autonomy and Care for dependent persons created a new subjective citizenship right in the Spanish National Territory: The right of those person who cannot take care of themselves, to receive the necessary attention by the public authorities. Likewise, the Act created the Autonomy and Dependency Care System (SAAD as per its Spanish acronyms) intended for the collaboration and contribution of all the Public Administrations involved which is also intended for the optimization of the public and private resources available. The Act includes three dependency stages and there shall be two levels in each of them depending on the autonomy of the persons and the specific care they require (there are specific provision for child under 3 years of age).

In the Autonomous Community of Andalusia, it has been submitted 282,415 applications in May 2009, out of which, 226,636 (86.69%) of them have been ruled, and out of the later, 50.27% (113,923) corresponds to Dependency Stage III and 27.22% (61.688) out of them corresponds to Dependency Stage II [33].

This Act guarantees to the dependent persons and their caregivers, to have access to social services (home care assistance, day care centres, telecare, places in elderly nursing homes) or the financial benefits they might need, however, given the experience in different places as described above, as well as the social significance of this regulation, some doubts arise in regards to its possible effects in the physical and psychological health of the family caregivers.

From the point of view of the development of this incipient Act, as well as on the potential impact in the population’s health, to carry out studies which provides relevant information or which might assist to create hypothesis for the continuity of lines of research is an unavoidable need for the design of health and social policies in the next few years.

This study is intended to know how the Act on Promotion of Personal Autonomy and Care for dependent persons is affecting the mental health, quality of life and use of health services by the family caregivers and also, if the benefits stage chosen makes the difference in these variables.

## Methods/design

### Objectives

#### General objective

To know the possible effects of the benefits of the Act on Promotion of Personal Autonomy and Care for dependent persons (hereinafter referred to as LPAPAD as per its Spanish acronyms) on the mental health, quality of life and use of health services of the dependent’s family caregivers.

Main objectives:

1. To describe modifications in the mental health of the dependent’s family caregivers who received the benefits of the LPAPAD.

2. To describe modifications in the overburden of the dependent’s family caregivers who received the benefits of the LPAPAD.

3. To describe modifications in quality of life of the dependent’s family caregivers who received the benefits of the LPAPAD.

4. To describe modifications in the use of health services by the dependent’s family caregivers who received the benefits of the LPAPAD.

5. To compare the results achieved (mental health, overburden, quality of life and use of health services) in those family caregivers, according to the type of benefits chosen.

### Design

Longitudinal descriptive study, following-up a cohort. The arrangements for the application, assessment, resolution and final grant of the benefits included in the LPAPAD, which naturally generates a sole cohort in two phases: those receiving the benefits, and those who do not have access to such benefits. The initial phase includes approximately a period not lower than 6 months which allows the baseline assessment of certain interesting variables in the applicant caregivers who are free from the exposure factor (reception of any of the benefits modalities of the LPAPAD). Following, the cohort shall be followed up once they are immersed in the exposure factor of the benefits’ reception.

### Study population

#### Study population

Family caregivers relatives of dependent patients in Stage II and/or III who apply for Dependent Care Assistance in the districts of Malaga, Costa del Sol, Valle del Guadalhorce and Jaen Northeast district during 2011.

Inclusion criterion:

• Family caregivers of patients who have been recently included in the Dependency-Stage II and/or III, who apply for Dependent Care Assistance.

• Family caregivers of patients who were not included initially in Stage II and/or III and are included in such stage after checking their dependency stage (Social and Health Services).

• Existence of a reference family caregiver duly identified who will play the role of caregiver during the study.

• Acceptance to take part in the study.

Exclusion criterion:

• Caregivers who reject to take part in the study.

• Professional caregivers.

• Patients with dependency level not included in Stage II and/or III, otherwise, registering a Barthel index score above 60.

• Caregivers who do not expect to be in this situation in the next 12 months (whether for personal reasons or due to the patient situation: unfortunate prognosis, change of address, transfer of the caregiver role to another member of the family, etc.).

#### Sample size

The sample size has been estimated to consider all the variables, assuming an alpha value of 0.05, powered at 80% in all of them. The calculations were estimated with the Software STATA 10.0. A sample including 474 caregivers shall cover the sample requirements for all the objectives. This sample shall increase 20% to cover possible losses (n=569).

### Variables and measuring instruments

#### Outcomes

The mental health shall be explored by two groups of variables, namely, depression and anxiety. The measuring instruments for such purpose shall be the questionnaire on the health of the patient PHQ-9 and the Goldberg anxiety subscale, jointly with the existence or inexistence of medical and nursing diagnosis related with the problem assessed. The caregiver effort index shall be the measuring instrument and the existence or inexistence of nursing and medical diagnosis shall be taken into account. In regards to quality of life, the measuring instrument shall be the questionnaire on the quality of life SF-12.

In regards to the use of the health services, it will be assessed the use of Primary Health Care, outpatient hospital services, hospital admission and the consumption of analgesic, antidepressant and anxiolytics.

#### Exposure variable

This shall be measured taking into consideration whether they have received the assistance pursuant to the LPAPAD or not, during the studying period.

Socio-demographic and sample characterization variables: In this study, it shall be taken into account different factors as the age, sex, level of education, occupation, caregiver’s level of the income self-assessment, daily dedication to health care as well as it will be studied the age, sex, ability to carry out the basic activities in daily life (Barthel index) and cognitive impairment (Pfeiffer Assessments) of the person receiving health care.

#### Data collection

The caregivers shall be selected consecutively according to the application order for the dependency benefits in the different districts taking part in the study. The application received requires a health report which is carried out in the Health Centres by the nursing professional members and guarantees the collection of eligible persons without losses as well as the baseline assessment of all variables. It is estimated that the recruitment period of the cohort takes around 6 to 8 months. All the applications fulfilling the inclusion criterion shall be selected for the study.

#### Follow-up

The follow-up shall be carried out at 3, 6, 9 and 12 months when, the variables of the study which are related with the objectives thereof, shall be reassessed. The information shall be collected by the respective nurse. All the information shall be included individually in a data base and this way, it will just be displayed the number of user of the digital medical history of the Andalusian Public Health System (NUHSA as per its Spanish acronyms) as the sole identifying information. The follow-up at 6 months will be carried out upon the grant of the dependency benefits and shall be the first post-exposure follow-up.

#### Analysis

It will be carried out blind analysis by a tester independent from the research group.

Descriptive and exploratory analysis: It shall be carried out descriptive statistic of the variables achieving measures of central tendency and dispersion or percentages according to its nature and the normal distribution shall be assessed by means of the Kolmogorov-Smirnov test (K-S test) and the Shapiro-Wilk test. Likewise, it shall be also tested the distribution asymmetry and kurtosis. According to the symmetry, it will be carried out non-linear transformations of Tukey to improve this field. The baseline sample shall be stratified according to the differential values of the main variables on Mental Health, overburden, quality of life and use of health services, as well as socio-demographic factors (age, sex, level of education, etc.) in order to identify possible baselines differences. In this case, the analysis shall be carried out adjusted by those variables in which has been observed influence.

#### Bivariate analysis

It shall be carried out a comparative test by means of the chi-squared test and Mantel-Haenszel statistic tests, applying Fisher’s exact test, if this would be necessary in qualitative variables. The precision of all parameters shall be estimated by a 95% confidence interval calculation. In regards to continuous variables, it will be carried out a bivariate analysis by the Student-t for independent samples if these have a normal distribution. In the event that the distribution is not normal, it shall be applied non-parametric tests (U-Mann–Whitney test and Wilcoxon test).

#### Multivariate analysis

It shall be carried out a multivariate logistic/multinomial regression analysis (according to the variable analysed) in order to determine factors associated to the modification of the variables of interest. It is not ruled out the construction of a Cox regression model for mental health, overburden, and establishment of the corresponding hazard ratio according to predictor variables related to the type of assistance received and factors of the caregiver. In order to control possible confounding factors, it shall be applied the criterion of in regards to the data analysis [34].

### Limitations

As it is a descriptive study, the causality among the factors analysed could not be determined, despite the longitudinal design and the specific characteristics involved in the project, might create necessary conditions to generate verifiable hypothesis in prospective studies.

#### Social class

These variables shall not be applied in caregivers given that the standard classification available does not include unemployed persons and those persons older than 65, therefore, in such case, a large number of persons would not comply with the requirements to be included in the study.

#### Income level

We do not count on objective data in regards to the income level, therefore, the caregivers shall be asked for this information at the beginning of the study before the reception of any benefit or assistance. It is possible that the information provided is biased information derived from potential conflict of interest with the benefits to be received. However, different variables shall be taken into account as it is the level of education and the professional occupation.

### Ethic aspects of the research

The study has been reviewed and approved by the Ethics and Health Research Committees of the districts of Malaga, Costa del Sol, Northeast Jaen and clinical research subcommittee of the University Hospital Virgen de la Victoria in Malaga.

The guidelines of Good Clinical Practice have been applied at all times as well as the ethics principles established for medical research involving human subjects pursuant to the Declaration of Helsinki and further amendments. The clinical information is isolated from the identification information and the data bases are encrypted and under custody in specific computers solely used for this project. All the registries are carried out pursuant to the legislation in force in regards to personal data protection. Each participant must sign the informed consent document in order to be part in this study.

This research study has been financed by *Instituto de Salud Carlos* III (Health Institution Carlos III), part of the Ministry of Economy and Finance of the Spanish Government and the Department of Health and Social Welfare of the Government of Andalusia. The commencement date of the study was January 2011 and will last three years, therefore, it is foreseen the study concludes on December 2013.

## Discussion

In Spain, the Act on Promotion of Personal Autonomy and Care for dependent persons created a new subjective citizenship right in the Spanish National Territory: The right of those person who cannot take care of themselves, to receive the necessary attention by the public authorities.

This Act guarantees to the dependant persons and their caregivers, the access to social services (home care assistance, Day Care Centres, telecare, places in homes for elderly persons) or the benefits they might need, however, given the experience in different environments, as well as the social implication of this Act, some question arises in regards to the possible effects in the physical and mental health in the caregivers. The potential benefit of registering in Andalusia the possible short-term effects of the Act LPAPAD, which is the main agent supporting the dependent persons (and their caregivers), will help in the decision-making process on social and health policies as well as on a possible reorientation of services or which might lead to the development of the Act.

## Abbreviations

LPAPAD: Act 39/2006 dated December 14^th^, on Promotion of Personal Autonomy and Care for dependent persons (LPAPAD as per its Spanish acronyms).

## Competing interests

The authors state there is no conflict of interest.

## Authors’ contributions

MCFG has taken part in the creation and design of the study and drafted the first version of the study, as well as the manuscript. JMMA, FJMS, RCA, ECF, JPBS, FJNM, ILA, MCM, CBN have taken part in the creation and design of the study and they also critically reviewed the draft of the manuscript, providing a key intellectual contribution in the final version. All the authors herein have read and approved the final manuscript.

## Pre-publication history

The pre-publication history for this paper can be accessed here:

http://www.biomedcentral.com/1472-6963/12/462/prepub
